# Influence of Demographic Factors on Presentation, Symptoms and Function in Carpal Tunnel Syndrome

**DOI:** 10.3390/healthcare14172817

**Published:** 2026-09-02

**Authors:** Justyna Napora, Pawel Korolkiewicz, Agnieszka Blacha, Agata Kreńska, Tomasz Mazurek

**Affiliations:** Department of Orthopaedics and Traumatology of the Locomotor System, Medical University of Gdansk, 80-210 Gdansk, Poland

**Keywords:** carpal tunnel syndrome, carpal tunnel release, QuickDASH, BCTQ, demographic factors

## Abstract

**Background:** Carpal tunnel syndrome (CTS) is the most common peripheral nerve entrapment disorder and a leading cause of pain, sensory disturbances, and functional impairment of the hand. Although carpal tunnel release (CTR) is an established treatment, the influence of demographic characteristics on symptom severity and postoperative functional recovery remains incompletely understood. **Aim:** This study assessed short-term functional improvement following carpal tunnel release and explored associations between demographic characteristics and patient-reported outcomes before and after surgery. **Materials and Methods:** Thirty-two adults with electrophysiologically and ultrasonographically confirmed CTS underwent minimally invasive carpal tunnel release. Functional outcomes were assessed preoperatively and three months postoperatively using the Quick Disabilities of the Arm, Shoulder and Hand (QuickDASH) questionnaire and the Boston Carpal Tunnel Questionnaire (BCTQ). Demographic variables, including sex, education, occupation, place of residence, symptom duration, dominant hand, and primary presenting complaint, were analysed using non-parametric statistical methods. **Results:** Significant postoperative improvement was observed in both outcome measures. Mean QuickDASH scores improved by approximately 54.2%, while total BCTQ scores improved by approximately 42% (both *p* < 0.001). Most demographic variables showed no statistically significant association with postoperative outcomes. Sex was significantly associated with both preoperative and postoperative QuickDASH scores, while the symptomatic hand was significantly associated with preoperative QuickDASH scores. No statistically significant associations were identified between the analysed demographic variables and BCTQ scores. Twenty-six patients (81.2%) reported clinical improvement. **Conclusions:** Minimally invasive carpal tunnel release was associated with substantial short-term improvement in symptoms and hand function at three months. Exploratory analyses identified statistically significant associations between selected demographic characteristics and patient-reported outcomes; however, the limited subgroup sizes preclude definitive conclusions regarding independent demographic effects. Larger cohorts with longer follow-up and multivariable analysis are required to confirm these findings.

## 1. Introduction

Carpal Tunnel Syndrome (CTS) is the most common nerve entrapment syndrome worldwide [1]. Compression of the median nerve results in a wide range of symptoms, including pain, paraesthesia, and weakness within the median nerve distribution of the hand [2]. CTS is more prevalent in women than in men, particularly between 40 and 60 years old [3,4]. Professionally active adults, particularly those performing repetitive wrist movements, are at increased risk of developing CTS [5,6,7,8]. Presentation to an orthopaedic outpatient clinic represents an important stage in the diagnosis and management of CTS. Several factors influence when and how patients present for specialist assessment, including socioeconomic status. Studies have shown that wealthier patients tend to present earlier and are more likely to adhere to postoperative rehabilitation protocols [9,10]. This inequality is also exacerbated by the urban–rural disparity in access to healthcare. Together, these factors contribute to considerable heterogeneity within the CTS patient population [11].

Diagnosis of CTS is based on clinical symptoms, physical examination, and, when indicated, confirmatory investigations including electrodiagnostic studies, ultrasonography, MRI, and CT [12].

Management of CTS includes both conservative and surgical options, often depending on the severity of symptoms, effectiveness of nonsurgical treatment, patient condition, and physician and patient preference [13].

Surgical treatment has consistently been shown to provide greater symptomatic improvement than conservative treatment and to improve function of the affected hand [13,14,15]. Different techniques can be used to perform carpal tunnel release (CTR), the most common being open carpal tunnel release (OCTR). CTR may be executed as an ambulatory or inpatient procedure [16,17].

The primary objective of this study was to assess changes in patient-reported symptoms and upper-limb function three months after carpal tunnel release. A secondary exploratory objective was to examine associations between demographic and clinical characteristics and preoperative or short-term postoperative outcomes. Previous studies have suggested that demographic variables such as age, sex, occupation, socioeconomic status, and educational level may influence symptom severity, treatment-seeking behaviour, and postoperative recovery [5,6,7,8,9,10,11]. However, published findings remain inconsistent, and many studies focused on individual demographic factors rather than evaluating multiple variables simultaneously. Consequently, further investigation into the relationship between demographic characteristics and patient-reported outcomes following CTR is warranted.

## 2. Materials and Methods

The inclusion criteria for this study were consecutively sampled adult patients who reported to the outpatient clinics of the authors with symptomatology consistent with CTS. Patients with bilateral CTS were included using only the more symptomatic limb. The exclusion criteria for this study were any systemic diseases/infections that influence the neurovascular system (i.e., diabetes), any current localised disease, infection, injury or prior surgery in the region of the wrist and hand. All patients who did not display characteristics of CTS in ultrasound and ENG were also excluded.

All patients underwent a comprehensive clinical assessment including medical history, physical examination, ultrasonography and electrophysiological testing. The ultrasound was performed at the level of the carpal tunnel entrance and at the level of the Pronator Quadratus muscle with a standard linear transducer with the patient’s wrist in a neutral position, enabling visualisation of the median nerve [18]. The ENG examination was done by measuring the amplitude and velocity at the level of the Abductor Pollicis Brevis and the II and III digits.

The operative treatment was performed in an ambulatory surgery centre. Regional intravenous anaesthesia (Bier block) was administered by a consultant anaesthesiologist [19]. A tourniquet was placed on the arm prior to surgery. Standard sterile surgical technique was maintained throughout the procedure. A minimally invasive, 1.5 cm incision was made above the flexor retinaculum. Under visual guidance, with minimal soft-tissue dissection, the transverse carpal ligament was cut [17]. No postoperative splinting was used. Patients were instructed to wear a sling, avoid strenuous activity for two weeks after surgery and use OTC painkillers as needed. Rehabilitation was recommended starting two weeks after surgery following consultation with a rehabilitation specialist. Patients were instructed to attend postoperative follow-up visits with the surgeon performing the procedure.

All patients were asked about demographic data, including age, sex, level of educational qualifications, size of settlement in which they resided, occupation, their dominant hand, the length of symptoms prior to presentation and the primary presenting complaint.

Several validated questionnaires were used to assess the functional capabilities of the patients. The Quick Disabilities of the Arm, Shoulder and Hand (QuickDASH/QD) questionnaire is an 11-item questionnaire that assesses the extent to which symptoms limit activities of daily and professional life on a Likert scale (1–5), with a higher score indicating more severe limitations [20].

The Boston Carpal Tunnel Syndrome Questionnaire (BCTQ) is a 19-item questionnaire targeted at patients with CTS to assess the severity of their functional limitation. It is divided into an 11-item Symptom Severity Scale (SSS), which asks the patient to assess the frequency and severity of symptoms such as pain and paraesthesia, and a Functional Status Scale (FSS), which asks about 8 common activities of daily living, such as bathing, dressing and household chores and whether these activities are limited by CTS symptoms. Both parts are assessed on a Likert scale (1–5), with a higher score indicating more severe limitations.

The patients were asked to fill out both questionnaires on the day of surgery and on the follow-up visit at 3 months postoperatively, independently, with clarification provided when requested [21]. All patients were able to complete the full follow-up protocol and complete the study.

The data, once collected, were anonymised and processed using Microsoft Excel^©^. Statistical analyses were considered exploratory because of the limited cohort size and the small and unequal numbers within several demographic subgroups. Given the distributional characteristics of the outcome data and the limited subgroup sizes, non-parametric methods were used for group comparisons. Normality was assessed using the Shapiro–Wilk test. Differences between paired preoperative and postoperative outcomes were assessed using the Wilcoxon signed-rank test. Comparisons between two independent demographic groups were performed using the Mann–Whitney U test, whereas variables with more than two categories were analysed using the Kruskal–Wallis test. All tests were two-sided, and a *p*-value < 0.05 was considered statistically significant. Non-significant subgroup findings were not interpreted as demonstrating absence of an association.

The study was approved by the bioethical committee of the Medical University of Gdansk prior to its commencement, number NKBBN/815/2022. Participation was unincentivized and voluntary, and patients were informed of their participation, purpose and use of personal data. Written and oral consent were obtained from every participant.

## 3. Results

Thirty-two patients were qualified for the study, with a predominance of female participants. Patient age ranged from 27 to 74 years, with a mean age of 53 years, with the largest proportion of patients being in their fifth decade of life. The majority of participants had middle or higher education, and 18 patients reported their work as primarily of a physical nature. Table 1 summarises the demographic distribution in more detail.

Table 2 illustrates the demographic categories and presents the mean values of the QuickDASH Score before operation, postoperatively, as well as the percentage difference between the two values. All demographic subgroups demonstrated improvement and a decrease in the QD score after the operation.

**Figure 1:**
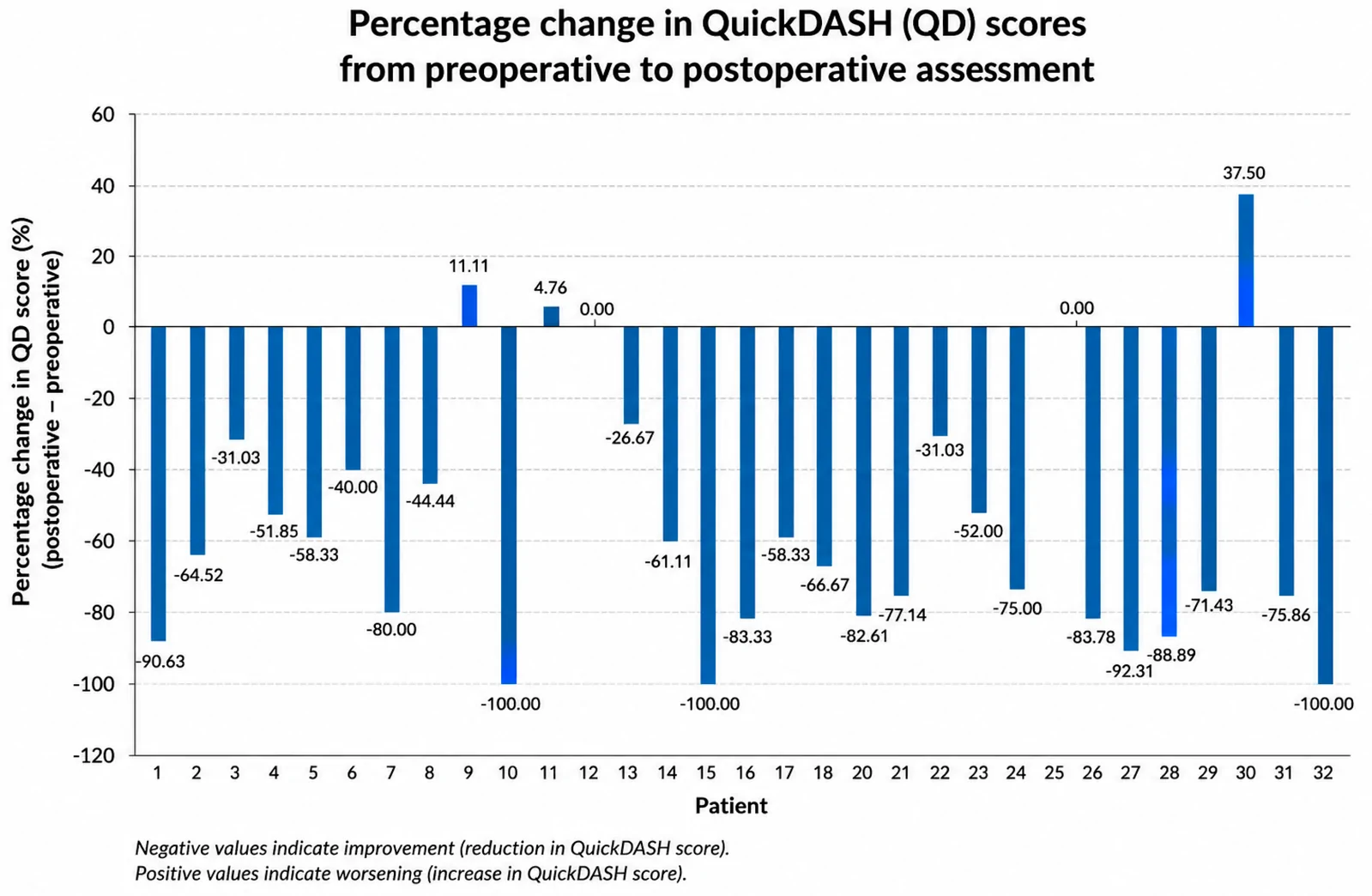
Percentage difference for pre- and postoperative QD scores for individual patients. Negative numbers indicate a decrease in QD, and therefore an improvement in hand function.

Six out of thirty-two (18.8%) participants of the study group showed no improvement in their QD scores after surgical intervention, with 3 participants reporting worsening limb function. Figure 1 demonstrates that on average, the mean QuickDASH score decreased by approximately 54%, from 53.95 points to 22.18 points.

Table 3 demonstrates the same demographic parameters with the mean values of the BCTQ. Similarly to the QuickDASH, all demographic subgroups showed significant improvement and a decrease in both subparts by approximately 43.38%. The mean scores dropped from 59.97 to 34.28 points, with the subscales dropping from 36.56 to 20.43 points in the SSS and from 23.31 to 13.84 points in the FSS subcategories.

Table 4 presents the statistical comparisons between demographic variables and preoperative, postoperative, and change scores for QuickDASH and BCTQ.

Only 6 patients reported no improvement or worsening of symptoms at the follow-up. Five of those patients reported incomplete adherence to postoperative recommendations or rehabilitation; however, these observations were descriptive and were not formally analysed. In the remaining patient, the lack of improvement was unable to be explained.

The Wilcoxon signed-rank test demonstrated significant improvements in both QuickDASH and BCTQ scores following surgery (both *p* < 0.001). In the subgroup analyses, sex was significantly associated with both preoperative (*p* = 0.028) and postoperative (*p* = 0.013) QuickDASH scores. In addition, the symptomatic hand was significantly associated with preoperative QuickDASH scores (*p* = 0.042), whereas no significant association was observed for postoperative QuickDASH scores (*p* = 0.137). No statistically significant associations were identified between education level, place of residence, occupation, symptom duration, or presenting complaint and QuickDASH scores (*p* > 0.05). For the BCTQ, none of the analysed demographic variables demonstrated statistically significant associations with either preoperative or postoperative scores or with the magnitude of postoperative improvement (all *p* > 0.05), although the association between sex and postoperative BCTQ scores approached statistical significance (*p* = 0.051).

## 4. Discussion

Overall, the findings demonstrate that CTR is an effective method of reducing symptomatology and improving hand function at three months post-surgery. The questionnaires assessing function showed improvements of 42% and 54%, respectively, which were shown to be statistically significant.

Gender is a significant risk factor for CTS. The disease affects women several times more often than men. Middle-aged women are most often at risk [8,11,22]. In the present study, sex was significantly associated with both preoperative and postoperative QuickDASH scores. Women demonstrated higher mean preoperative disability scores than men and also greater absolute postoperative improvement (60.01% vs. 39.26% in the QD, 46.35% vs. 29.20% in the BCTQ in favour of women). However, no statistically significant association between sex and BCTQ scores was identified, although the postoperative comparison approached statistical significance. These findings should be interpreted cautiously because of the relatively small cohort and the unequal distribution of male and female participants. Other authors have noticed smaller or non-significant differences between sex [23]. Several explanations may account for why women responded better to the CTR, such as smaller anatomy, stronger adherence to postoperative recommendations or the prevalence of female subjects in the study. Other authors present varying conclusions, which would require further research in the future.

Most cases of carpal tunnel syndrome are idiopathic, but physical work predisposes one to the condition [1,3,5]. Mechanical interaction related to the presence of compressive forces on the median nerve explains the biomechanical risk factors associated with the development of carpal tunnel syndrome [7]. Risk factors for carpal tunnel syndrome include a forceful grip, wrist extension and flexion, and exposure to vibration. Occupations requiring repetitive tasks and strenuous manual work can exacerbate the compression of the nerve in the carpal tunnel. People working in such professions are susceptible to cumulative micro-injuries that contribute to the development of carpal tunnel syndrome [6,12,24]. Over half of the patients in the present study reported to be working physically. Contrary to existing literature, there was no observable significant difference pre- and postoperatively between patients with different types of work. There was also no statistically significant improvement between pre- and postoperative function when comparing physically working individuals vs. non physically working ones, and even those who did not engage in any type of professional activity.

Residents of rural areas have limited access to healthcare due to the limited number of medical services provided in these areas. The lack of orthopaedic surgeons in rural areas is due to issues such as an ageing medical workforce, fewer students from rural areas, and limited local healthcare options [25]. A referral is required to see an orthopaedic surgeon, so access to orthopaedic services is also linked to access to family doctors. Significant disparities in access to both family doctors and orthopaedists exist in rural areas [26]. This factor proved to be statistically insignificant in this case for all measurements, contrary to most literature. One possible explanation is the overwhelming number of participants from large cities, skewing the results. A further possibility is the fact that the region of northern Poland, where the study was conducted, remains one of the wealthiest and best-connected regions in the country, where access to orthopaedic services is less problematic than in other regions.

An interesting observation was the significant association between the symptomatic hand and preoperative QuickDASH scores. However, this association was no longer statistically significant following surgery, suggesting that postoperative functional improvement was comparable regardless of the affected side. Those observations are debated in the orthopaedics community, with some authors claiming that the risk of CTS is greater in left-handed individuals, whilst others claim no observable differences [27,28]. A more probable explanation is the fact that the QD scores are significantly lower in the nondominant hand [27]. In this study, over 31% of the participants reported symptoms of CTS in their left hand, whilst the percentage of left-handed people in the population is estimated at around 10%.

In a study by Yucel et al., 89 patients who had undergone open carpal tunnel release surgery were evaluated to determine which hand-specific questionnaire was the most effective. They compared BCTQ, MHQ, QuickDASH and the DHI in patients with carpal tunnel syndrome. The QuickDASH questionnaire demonstrated a significant correlation with pain intensity, paraesthesia and BCTQ scores (*p* < 0.001). The assessment tools that were analysed demonstrated mutual consistency. It appears that QuickDASH is a more functional and practical tool for the assessment of patients following surgical release of the CTS [29].

Moradii et al. in their study showed that the Disabilities of the Arm, Shoulder and Hand (DASH) questionnaire, which was developed two decades ago, does not need to be updated with daily activities resulting from technological advances. The updated QuickDASH questionnaire showed a notably lower level of reported disability compared to the standard version; however, the 4-point difference (33 vs. 37) may not be clinically significant [30].

Several limitations should be considered when interpreting the present findings. First, the relatively small cohort limits the precision and statistical power of the demographic subgroup analyses. Although all 32 patients contributed paired preoperative and postoperative measurements to the principal analysis, subdivision according to demographic characteristics produced small and, in several instances, markedly unequal groups. Consequently, these secondary analyses should be regarded as exploratory and are principally capable of detecting relatively large between-group differences. Small or moderate demographic associations may therefore have remained undetected, and non-significant findings should not be interpreted as evidence of absence of an association.

The limited number of observations also precluded reliable simultaneous adjustment for multiple demographic and clinical covariates. Accordingly, the subgroup analyses are unadjusted and hypothesis-generating, and confirmation of independent demographic associations requires a larger cohort.

Second, the three-month postoperative assessment represents an evaluation of early symptomatic and functional outcome rather than definitive long-term neurological recovery. Recovery may continue beyond this period, and the present study cannot determine the ultimate magnitude or durability of treatment effect. Longer prospective follow-up is therefore required.

Finally, the single-centre design and limited representation of several demographic categories restrict the generalizability of the subgroup findings. Larger, preferably multicentre studies are required to validate the observed associations.

Another possibility for improvement would have been the stratification of CTS symptomatology, as it is possible that QD by itself did not accurately portray the severity of the disease. It must be taken into consideration that BCTQ may have some limitations. Schulze et al. and Multanen et al. showed in their study that BCTQ has acceptable construct validity, although gender differences at some ages were noticed [31,32].

## 5. Conclusions

Carpal tunnel release was associated with substantial improvement in patient-reported symptoms and upper-limb function at three months. Exploratory analyses identified potential associations between selected demographic characteristics and patient-reported outcomes; however, the limited and unequal subgroup sizes prevent definitive conclusions regarding independent demographic effects. These findings should therefore be considered hypothesis-generating. Larger prospective studies with longer follow-up and sufficient sample size for multivariable adjustment are required to determine whether demographic characteristics independently influence outcome following carpal tunnel release.

## Figures and Tables

**Table 1:** Demographic distribution of the study group.

		n = 32	%
Sex			
	Male	9	28.13
	Female	23	71.88
Education			
	Basic	2	6.25
	Professional	8	25.00
	Middle	12	37.50
	Higher	10	31.25
Size of place of residence			
	Village	6	18.75
	Small/middle sized town	4	12.50
	Large town/city	22	68.75
Type of profession			
	Physical	18	56.25
	Sedentary	6	18.75
	Non-working	8	25.00
Symptomatic Hand			
	Left	10	31.25
	Right	22	68.75
Duration of symptoms			
	<6 months	4	12.50
	≤1 year	5	15.63
	≤2 years	8	25.00
	2–>5 years	12	37.50
	>5 years	3	9.375
Compaints			
	Pain, paraesthesia	24	75.00
	Functional limitations	8	25.00

**Table 2:** Demographic differences in mean QD score pre- and postoperatively.

		Quick DASH Before	SD (±)	Quick DASH After	SD (±)	Absolute Difference	% Difference
Sex							
	Male	52.53	21.20	27.78	10.81	24.75	39.26
	Female	56.82	15.44	20.95	17.10	35.87	60.01
Education							
	Basic	61.36	16.60	34.09	11.47	27.27	44.44
	Professional	57.07	20.07	29.55	13.75	27.52	37.97
	Middle	53.22	15.26	18.56	19.77	34.66	64.36
	Higher	56.59	13.53	20.91	14.05	35.68	57.52
Size of place of residence							
	Village	63.26	14.44	31.44	11.47	31.82	50.66
	Small/middle sized town	60.23	20.27	26.14	10.98	34.09	47.78
	Large town/city	52.69	16.62	19.94	17.90	32.75	56.30
Type of profession							
	Physical	59.34	16.30	22.98	18.22	36.36	55.23
	Sedentary	53.79	19.74	20.83	14.06	32.96	60.38
	Non-working	48.58	18.14	24.15	14.47	24.43	47.15
Symptomatic Hand							
	Left	59.32	22.67	19.55	19.05	39.77	64.65
	Right	53.93	14.17	24.38	15.55	29.55	49.41
Duration of symptoms							
	<6 months	55.61	21.27	22.87	25.13	32.74	54.18
	≤1 year	55.61	20.37	29.55	9.18	26.06	41.71
	≤2 years	50.85	20.37	19.89	18.02	30.96	39.26
	2–>5 years	56.06	19.36	17.99	15.93	38.07	59.16
	>5 years	50.00	9.46	24.24	16.12	25.76	50.06
Complaints							
	Pain, paraesthesia	54.64	18.08	26.33	17.49	28.31	46.88
	Functional limitations	58.52	14.41	12.50	9.75	46.02	76.07

**Table 3:** Mean score for total BCTQ for each demographic group. (Please note that the negative % difference shows an improvement in the score.)

		BCTQ Pre-OP	SD (±)	BCTQ Post-OP	SD (±)	Absolute Difference	% Difference
Sex							
	Male	59.00	6.22	41.25	12.03	17.75	−29.20
	Female	60.78	8.41	32.29	10.32	28.49	−46.35
Education							
	Basic	54.00	8.71	32.00	8.01	22.00	−40.74
	Professional	60.43	6.45	46.71	16.02	13.72	−22.73
	Middle	60.16	10.86	34.17	8.93	25.99	−41.71
	Higher	59.40	4.43	31.60	6.65	27.80	−46.98
Size of place of residence							
	Village	59.40	8.81	31.60	6.89	27.80	−46.67
	Small/middle sized town	62.25	3.30	40.00	23.56	22.25	−36.57
	Large town/city	60.50	8.24	34.55	9.33	25.95	−42.04
Type of profession						0.00	
	Physical	58.65	8.07	37.18	13.56	21.47	−36.09
	Sedentary	61.67	9.79	28.33	9.22	33.34	−53.25
	Non-working	61.13	4.96	31.50	9.13	29.63	−48.61
Symptomatic Hand							
	Left	63.20	9.42	29.90	6.36	33.30	−52.48
	Right	59.10	6.85	36.76	13.11	22.34	−37.39
Duration of symptoms							
	<6 months	60.16	5.60	34.35	12.84	25.81	−42.35
	≤1 year	61.40	2.59	28.20	19.62	33.20	−53.88
	≤2 years	57.63	10.10	31.63	8.60	26.00	−43.26
	2–5 years	61.64	9.16	35.18	12.40	26.46	−42.95
	>5 years	54.00	4.73	32.00	2.65	22.00	−40.74
Complaints						0.00	
	Pain, paraesthesia	59.61	7.67	35.43	10.81	24.18	−38.88
	Functional limitations	60.63	8.33	27.00	14.88	33.63	−55.14

**Table 4:** *p*-Values for comparisons between demographic variables and preoperative, postoperative, and pre-versus postoperative QuickDASH and BCTQ scores.

	Test Used	Quick Dash Pre-OP	Quick Dash Post-OP	Pre vs. Postop	BCTQ Pre-OP	BCTQ Post-OP	Pre vs. Postop
Sex	Mann–Whitney U	0.675	0.051	0.367	0.705	**0** **.013**	**0.028**
Education	Kruskal–Wallis	0.266	0.324	0.331	0.933	0.075	0.051
Size of place of residence	Kruskal–Wallis	0.131	0.28	0.127	0.383	0.668	0.626
Type of profession	Kruskal–Wallis	0.552	0.439	0.448	0.356	0.520	0.231
Symptomatic Hand	Mann–Whitney U	0.392	0.596	0.464	0.214	0.137	**0.042**
Duration of symptoms	Kruskal–Wallis	0.151	0.758	0.280	0.607	0.554	0.527
Complaints	Mann–Whitney U	0.473	0.76	0.948	0.647	0.122	0.107

All *p*-values < 0.05 have been highlighted in bold.

## Data Availability

The data presented in this study are available on request from the corresponding author due to privacy restrictions.
